# Daily Protein and Energy Intake Are Not Associated with Muscle Mass and Physical Function in Healthy Older Individuals—A Cross-Sectional Study

**DOI:** 10.3390/nu12092794

**Published:** 2020-09-12

**Authors:** Grith Højfeldt, Yusuke Nishimura, Kenneth Mertz, Simon R. Schacht, Jonas Lindberg, Mikkel Jensen, Morten Hjulmand, Mads Vendelbo Lind, Tenna Jensen, Astrid Pernille Jespersen, Soren Reitelseder, Inge Tetens, Lars Holm

**Affiliations:** 1 Institute of Sports Medicine Copenhagen, Department of Orthopaedic Surgery M, Bispebejrg Hospital, 2400 Copenhagen, Denmark; grithwh@gmail.com (G.H.); shotmertz@hotmail.com (K.M.); lindberg.jonas90@gmail.com (J.L.); m.jensen89@live.dk (M.J.); mortenhjulmand1@gmail.com (M.H.); s.reitelseder@gmail.com (S.R.); L.Holm@bham.ac.uk (L.H.); 2 School of Sport, Exercise and Rehabilitation Sciences, University of Birmingham, Edgbaston, Birmingham B15 2TT, UK; 3 Department of Nutrition, Exercise and Sports, University of Copenhagen, 1958 Copenhagen, Denmark; simonschacht@nexs.ku.dk (S.R.S.); madslind@nexs.ku.dk (M.V.L.); ite@nexs.ku.dk (I.T.); 4 Copenhagen Centre for Health Research in the Humanities, Saxo Institute, University of Copenhagen, 2300 Copenhagen, Denmark; tennaje@hum.ku.dk (T.J.); apj@hum.ku.dk (A.P.J.); 5 Department of Biomedical Sciences, Faculty of Health and Medical Sciences, University of Copenhagen, 2200 Copenhagen, Denmark

**Keywords:** sarcopenia, ageing, dietary protein, protein distribution, elderly, muscle mass

## Abstract

Dietary protein has a pivotal role in muscle mass maintenance with advancing age. However, an optimal dose and distribution of protein intake across the day as well as the interaction with energy intake for the maintenance of muscle mass and physical function in healthy older adults remain to be fully elucidated. The purpose of this study was to examine the association between muscle mass, strength, and physical function, and the total amount and distribution of protein and energy intake across the day in healthy older individuals. The research question was addressed in a cross-sectional study including 184 Danish men and woman (age: 70.2 ± 3.9 years, body mass: 74.9 ± 12.1 kg, Body Mass Index (BMI): 25.4 ± 3.7 kg/m^2^) where a 3-day dietary registration, muscle mass, strength, and functional measurements were collected. We found that neither daily total protein intake nor distribution throughout the day were associated with muscle mass, strength, or physical function. Consequently, we do not provide an incentive for healthy older Danish individuals who already adhere to the current internationally accepted recommended dietary protein intake (0.83 g/kg/day) to change dietary protein intake or its distribution pattern throughout the day.

## 1. Introduction

Dietary protein continues to receive attention in an effort to combat sarcopenia [1,2,3,4,5]. The most recent recommendation from the Nordic Council of Ministers is 1.1–1.3 g protein/kg Body Weight (BW)/day (15–20 energy %) for older adults above the age of 65 years [6]. Meanwhile, globally, the recommended intake for all adults remains at 0.83 g protein/kg BW/day [7,8]. The global recommendation was developed based on a meta-analysis of nitrogen balance measurements from various dietary protein intake levels [9]. However, the recommended value was based on nitrogen balance methodology that, with currently available data, neither distinguishes age nor accounts for factors such as sex and meal distribution patterns across the day. While some studies report similar protein requirements in healthy younger and older adults using the nitrogen balance methodology [10,11], a growing number of epidemiological studies assessing the associations between protein intake and muscle mass suggest that muscle mass and/or function in older individuals can be maintained with protein intake levels matching the recommendation made by the Nordic Council of Ministers [12,13,14,15,16].

Protein distribution throughout the day is considered an important factor in muscle mass maintenance [17,18,19,20]. This notion has grown from the findings of a marked, transient stimulation of Muscle Protein Synthesis (MPS) in response to hyperaminoacidemia [21,22] by the bolus intake of fast absorptive proteins [23]. The dose–response relationship between protein intake and MPS plateaus at ~30–45 g protein per serving (0.4–0.6 g/kg BW) for a 75 kg older adult (>65 years) [23] with no cumulative effect of protein intake over a period of time and rather oxidized beyond this amount [24]. This has led to the concept of an optimal dose of ~30 g of protein per meal to maximally stimulate MPS [23] which is required at all three main meals and snacks in a day (1.2–1.8 g/kg BW/day) [25,26]. However, some challenges exist when daily protein recommendations are extrapolated from MPS, which have been determined in acute experimental studies in response to various doses of protein ingestion. Among these, the interaction between protein and energy intake [27] as the intake of other macronutrients and/or total energy is known to markedly affect postprandial aminoacidemia [28,29,30] and whole-body net protein balance [31]. In addition, the type of studies investigating the stimulatory effect of a bolus ingestion of protein rarely account for the habituated level of dietary intake (i.e., the protein intake the participant is accustomed to). We and others have demonstrated that adaptation to higher protein intakes alters amino acid utilization via an increase in amino acid oxidation and urea production [32,33,34,35].

A cross-sectional study allows for an investigation of the association between habitual protein and energy intake in a matrix of normal foods and physiological outcomes, such as muscle mass, strength, and functional capabilities. In westernized and industrialized cultures, the common dietary pattern consists of three main meals throughout the day with smaller in-between snacks. A recent cross-sectional analysis including 38 older UK citizens above the age of 70 years found that more than half of total protein intake was consumed in one meal [36]. Similar dietary patterns have been reported in studies from westernized countries, showing that >43% of protein is ingested in one single meal [37,38]. If we accept the premise that there is a dose–response relationship between protein intake and net postprandial protein anabolism, the protein content per meal is important. Additionally, total daily protein intake and per meal protein requirement for the maintenance of whole-body protein are interrelated with energy intake [27,39,40] and energy balance [41]. Thus, optimal protein intake for the maintenance of muscle mass should be assessed in combination with energy intake [42].

The purpose of this cross-sectional study was to examine how daily protein and energy intake as well as distribution are associated with muscle mass, strength and physical function in healthy, older and well-functioning Danish men and women.

## 2. Materials and Methods

The results are based on a cross-sectional study of 184 older Danish home-dwelling men and women from the CALM (Counteracting Age-related Loss of skeletal Muscle mass) cohort, which is described in detail elsewhere [43]. Methods with relevance for this article are explained below.

### 2.1. Participants

A total of 184 men and women older than 65 years of age were included in this cross-sectional study. The participants were recruited through local newspapers, magazines, radio programs, social media, presentations at senior centers and public events. All participants were within the inclusion and exclusion criteria listed in the method paper [43] and deemed healthy, as assessed by a medical doctor based on blood samples and an oral interview. Participant data are presented in Table 1. Participants were informed of the study design, risks, and exclusion criteria prior to obtaining written consent. The study complied with the latest Declaration of Helsinki (7th version). Ethical approval was obtained through The Danish Regional Committees of the Capital Region on 4 July 2013 (number H-4-2013-070). The CALM intervention study was registered at Clinicaltrials.org as NCT02034760.

### 2.2. Dietary Records

A 3-day consecutive weighed dietary and liquid registration (Wednesday to Friday) was collected by following instructions from trained staff as described by Schacht et al. [44]. Trained staff entered the dietary records into the VITAKOST^TM^ (MADLOG Aps, Kolding, Denmark) program which uses the Danish Food Composition Databank (version 7.01; Søborg; Denmark) for calculating the nutrient intakes.

### 2.3. Physical Activity Level

The activity level of the participant was estimated by taping an activPal (activPal 3™, activPal 3c™, or activPal micro; PAL Technologies, Glasgow, UK) to the anterior surface of the thigh, which monitoring the steps and body (thigh) position over a 4-day period. Weekend days were always included.

Energy expenditure reflecting the average daily Metabolic Equivalent of Tasks (MET) was calculated based on the algorithms provided in the activPal software. Data are reported as the average daily step count and average daily METs.

### 2.4. Identification of Under- and Overreporters

Under- and overreporters of energy intake were identified based on the Goldberg cut-off, which is determined by the ratio between the Energy Intake and Basal Metabolic Rate (EI:BMR) [45]. The BMR was estimated via the Cunningham equation (Resting Energy Expenditure (REE, kJ/day) = 370 + 21.6 × fat free mass (Lean Body Mass; LBM) × 4.184) [46] multiplied by the physical activity level in each participant (METs) assessed by an activPal [47]. As described by Black [47] a cut-off <0.76 was defined as an underreporter and >1.24 as an overreporter. All statistical analyses were performed including and excluding under- and overreporters.

### 2.5. Coefficient of Variation for Protein Distribution

The evenness of protein distribution between main meals in the day was determined by calculating the Coefficient of Variation (CV) (Standard Deviation (SD) divided by the mean) of the protein in grams per main meal, covering breakfast, lunch and dinner for each participant as previously reported [18,36,48]. Thus, a lower CV indicates that protein was consumed more evenly across main meals compared to a higher CV.

### 2.6. Appendicular Lean Mass

As a marker of muscle mass, the Appendicular Skeletal Muscle Index (ASMI) was assessed by whole body Dual-energy X-ray Absorptiometry (DXA) scans, using the encore v.16 software (Lunar iDXA; GE Medical Systems, Pewaukee, WI, USA). The scans were performed in an overnight fasted and euhydrated state, with the participants refraining from strenuous physical activity for 48 h prior to the scans. Regions of interest were set based on the default definitions provided by the scanner software. The same examiner controlled the default positioning of all regions, which were adjusted slightly when appropriate to take into account the interindividual differences in body placement and body size. Appendicular lean mass was assessed by the sum of lean mass in the arm and leg regions, and the ASMI was calculated by dividing the appendicular lean mass by the height squared [49].

### 2.7. Muscle Strength and Functional Capability

The functional capacities of the participants were assessed by applied functional measures and strength measures. Detailed methods for these measurements can be found elsewhere [43,50]. The strength measures included: the dominant hand grip strength using a grip strength dynamometer (DHD-1 (SH1001); SAEHAN Corporation, Changwon City, Korea), Maximal Voluntary isometric Contraction (MVC) of the dominant m. quadricep muscle strength, measured at 70-degree flexion in a Kinetic Communicator (model 500-11, Kinetic Communicator; Isokinetic International, Chattanooga, TN, USA). The applied functional measures included a 30 s chair stand test and a 400 m gait test. In the 30 s chair stand test [51], the number of stands completed in 30 s from a seated position (seat height: 44.5 cm) with hands crossed across the chest were counted. The 400 m gait test was performed on a 20 m course with no helping remedies, instructing the participants to walk the 400 m as fast as possible without running [52].

### 2.8. Food Questionnaire

At baseline, the participants received a questionnaire containing a range of questions related to their food preferences and habits as well as lifestyle and dietary changes throughout life. The questionnaires combined basic socioeducational data, quantitative questions and quantifiable qualitative questions. For this article, 149 questionnaires have been screened for information on dietary changes in relation to retirement.

### 2.9. Groups Division

Adjusted Body Weight (aBW) was determined to give a BMI of either 22 or 27 for participants exhibiting a BMI below 22 or above 27, respectively (i.e., either 22 or 27 times height in meters squared) [38,53]. This was done to ensure that the energy and nutritional requirements are expressed relative to a body weight representing a body composition within a healthy range [54,55,56].

If participants did not report any intake at a meal, they received a zero in their energy intake, but they were excluded from the group mean for protein intake.

For data on total intake and distribution of dietary protein and energy, participants were divided into two groups: a lower (<0.83 g/kg aBW/day) and a higher (≥1.1 g/kg aBW/day) protein intake group. These were based on the current Recommended Daily Allowance (RDA) by the European Food Safety Authority (EFSA) [7] and World Health Organization (WHO)/Food and Agriculture Organization of the United Nations (FAO)/United Nations University (UNU) [8] at 0.83 g/kg/day for adults and the current fifth edition of Nordic Nutrition Recommendations 2012 [6] at 1.1–1.3 g/kg/day for older adults above the age of 65 years in men and women. Further, we also converted the absolute protein intake in g/kg/day to the energy % expression, as the relative energy contribution from protein intake of total energy intake.

For the comparison between total protein intakes and physical parameters (), participants were divided into 3 different total daily protein intake groups as follows: <0.83 g/kg aBW/day, ≥0.83–<1.1 g/kg aBW/day, ≥1.1 g/kg aBW/day in men and women. Associations between the distribution of protein intake and functional abilities were examined only within the groups ingesting a lower protein (<0.83 g/kg aBW/day) and those ingesting a higher protein (≥1.1 g/kg aBW/day) in order to investigate ‘extremes’ and exclude the middle group, where inherent variation of the food recording methodology and participants eating behavior may make the group-affiliation unreliable.

### 2.10. Statistical Analysis

The higher and lower protein intake differences of participant characteristics were assessed using an unpaired t test. A three-way mixed effects model (average daily protein intake levels × sexes × main meals) was used for energy and protein intake and energy% from protein intake. A two-way ANOVA (sexes × average daily protein intake levels) was used for the ASMI, grip strength, MVC, 400 m gate time, 30 s chair stand test and CV of protein intake. Turkey’s multiple comparisons test was used as a post hoc test. Person’s correlation coefficient was used to identify associations between protein and energy intake and associations between the CV of protein distribution and ASMI, grip strength, MVC, 400 m gait time, and 30 s chair stand test. Statistical significance was set at *p* < 0.05 (two-tailed). The statistical analyses were performed using Prism version 8.1.2 (GraphPad Software, San Diego, CA, USA).

## 3. Results

### 3.1. Participant Characteristics

A total of 184 participants (53% men and 47% women) with a 3-day weighed dietary record with muscle mass, muscle strength, and muscle function measurements at the time of study entry of the CALM study [43] were included in the present study. Table 1 shows the participant characteristics for all 184 participants included, as well as divides men and women into lower (<0.83 g/kg aBW/day) and higher (≥1.1 g/kg aBW/day) protein intake groups. Individuals with a protein intake that does not fit in the category ‘lower’ or ‘higher’ (i.e., ≥0.83–<1.1 g/kg aBW/day) were included into the ‘all’ group only.

The age of the participants ranged from 65 to 82 years with no significant difference between men (69.8 ± 3.9 years) and women (70.5 ± 4.0 years), and no significant difference between women with a higher and a lower protein intake. However, the men with a higher protein intake were significantly younger than the men ingesting a lower protein intake (*p* < 0.05). Within groups of men and women, there was no difference between groups with higher and lower protein intakes with regards to body weight, adjusted body weight, lean body mass (LBM), appendicular LBM and step counts.

Based on the Goldberg cut-offs [45], underreporters (22% (*n* = 41)) and overreporters (10% (*n* = 18)) were identified based on their reported energy intake. However, all statistical outcomes were identical irrespective of the inclusion or exclusion of under- and overreporters. Thus, all figures and statistical outcomes were illustrated including all participants (i.e., both underreporters and overreporters).

### 3.2. Total Daily Energy and Protein Intake and Distribution per Main Meal

**Figure 1:**
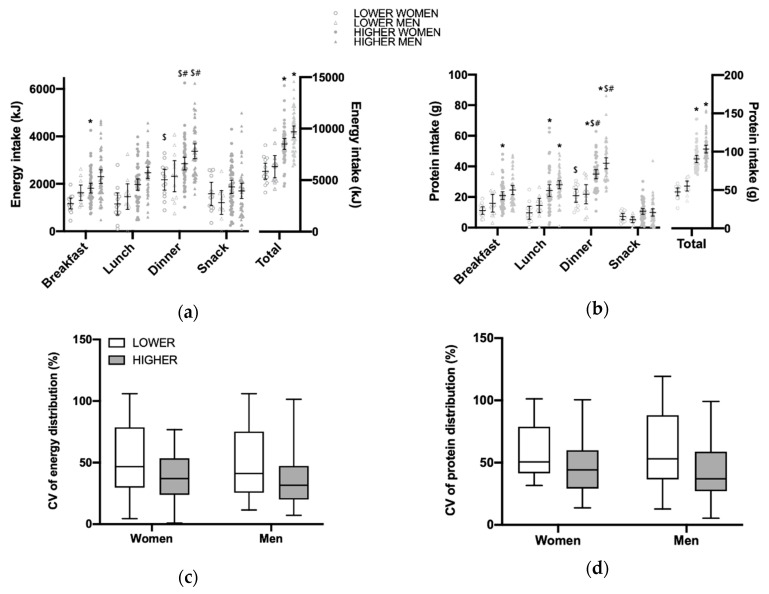
Baseline average energy (**a**) and protein (**b**) intake and distribution per meal, snacks during the day, and total daily intake. Turkey’s multiple comparison test (comparing between main meals and sexes). Values are means with 95% Confidence Interval(CI). Significance was set at *p* < 0.05. * indicates significant difference between the lower and higher protein intake in the same meal, $ indicates significant difference between breakfast and dinner, # indicates significant difference between lunch and dinner. The Coefficient of Variation (CV), as a measure of the distribution between the three main meals was shown for energy intake (**c**) and protein intake (**d**). For both energy and protein intakes, the participants were divided a lower (<0.83 g/kg Adjusted Body Weight (aBW)/day; *n* = 25, 12 men and 13 women) or higher (≥1.1 g/kg aBW/day; *n* = 98, 50 men and 48 women) protein intake. The boxes include the 25th, 50th, and 75th quartiles and whiskers represent the maximum and minimum values. Significance was set at *p* < 0.05. No main effect for intake amount or sex was found (*p* > 0.05).

In our cohort, participants consumed on average 82.8 ± 22.2 g protein/day corresponding to 1.13 ± 0.34 g protein/kg/day (men; 1.10 ± 0.31 g/kg/day, women; 1.16 ± 0.38 g/kg/day) (Table 1). The total daily energy intake and protein intake were greater in the higher protein intake group than the lower protein intake group for both men and women (right panel of Figure 1a,b). For both energy and protein intakes, there was a main effect between main meals (*p* < 0.0001). A post hoc test showed that in the higher protein intake group there was a higher protein intake at dinner compared to both at breakfast (*p* < 0.05) and at lunch (*p* < 0.05) for both men and women. In the lower protein intake group, energy and protein intakes at dinner were greater than at breakfast (*p* < 0.05) for women only. Main effects were also found for protein intake level between meals (*p* < 0.0001), with post hoc differences showing that women with a high protein intake had a higher total energy intake at breakfast, and a higher protein intake at all three main meals (*p* < 0.05). The men who had a higher protein intake only ingested more protein at lunch and dinner, compared to the men with a lower protein intake. Finally, there was main effect of sex (*p* = 0.01) for energy and protein intake.

The distribution of energy (Figure 1c) and protein (Figure 1d) expressed as the coefficient of variation (CV) between main meals did not differ between men and women nor between higher (42 ± 22 in men and 46 ± 20 in women) and lower (60 ± 34 in men and 59 ± 22 in women) protein intakes.

### 3.3. Daily Protein Intakes and the ASMI, Grip Strength, MVC, 400 m Gait Time, 30 s Chair Stand

**Figure 2:**
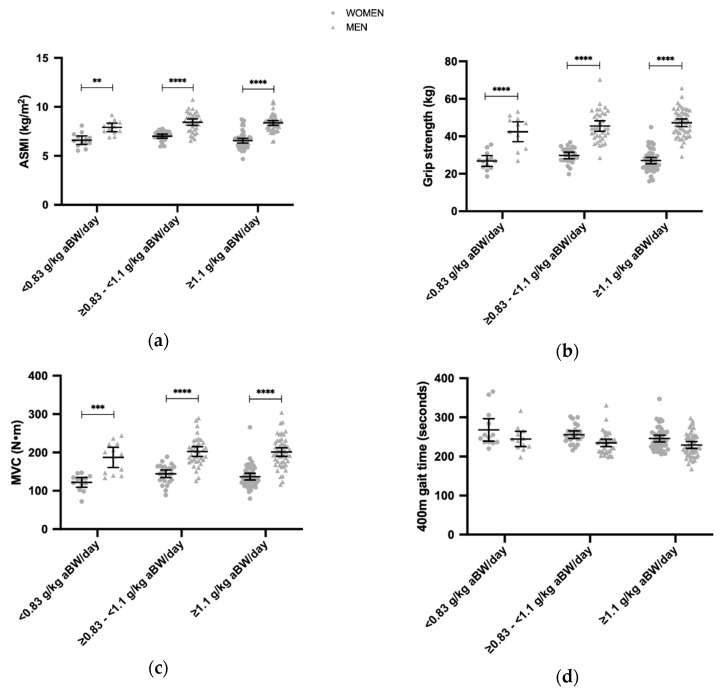

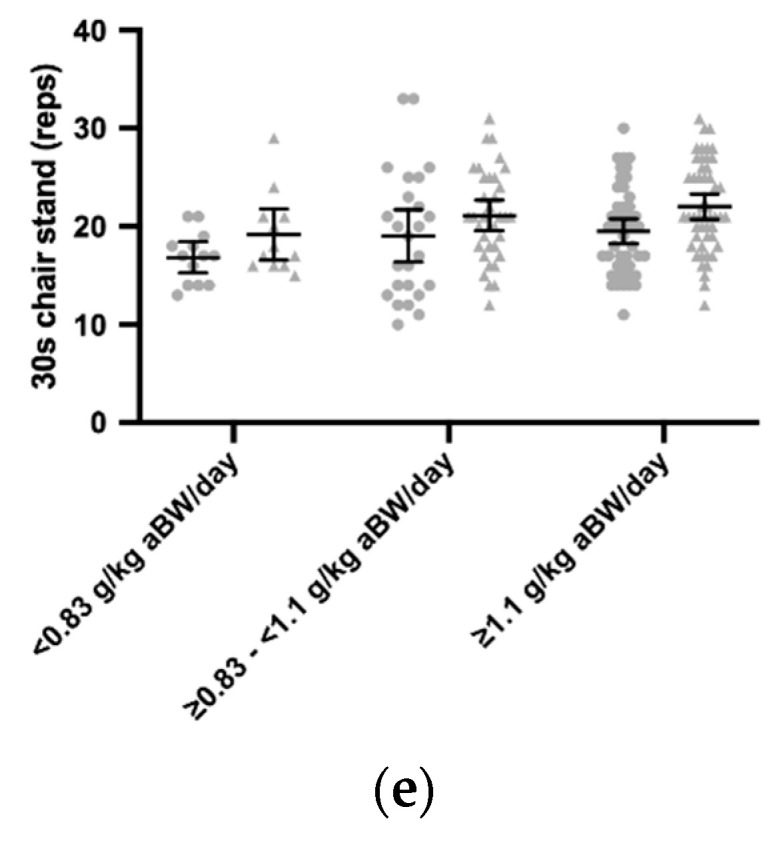
Appendicular Skeletal Muscle Index (ASMI) (**a**), grip strength (**b**), knee extension Maximal Voluntary isometric Contraction (MVC) (**c**), 400 m gait time (**d**), 30 s chair stand (**e**) divided into individuals with a protein intake of <0.83 g/kg aBW/day (*n* = 25, 12 men and 13 women), ≥0.83–<1.1 g/kg aBW/day (*n* = 61, 36 men and 25 women), ≥1.1 g/kg aBW/day (*n* = 98, 50 men and 48 women (*n* = 47 for MVC due to one missing value). Values are means with 95% CI. ** *p* < 0.01, *** *p* < 0.001, **** *p* < 0.0001, Turkey’s multiple comparison test (comparing between average daily protein intake levels and sexes). Significance was set at *p* < 0.05.

Overall, the ASMI, grip strength, MVC, 400 m gait time, 30 s chair stand did not differ between the three different protein intakes (<0.83 g/kg aBW/day, ≥0.83–<1.1 g/kg aBW/day, ≥1.1 g/kg aBW/day) in both men and women (Figure 2). However, sex differences were noted in the ASMI, grip strength, and MVC at <0.83 g/kg aBW/day, ≥0.83–<1.1 g/kg aBW/day, ≥1.1 g/kg aBW/day protein intake levels. The main effect of sex was identified in the ASMI (*p* < 0.0001), grip strength (*p* < 0.0001), and MVC (*p* < 0.01).

### 3.4. Associations between the Protein Distribution and ASMI, Grip Strength, MVC, 400 m Gait Time and 30 s Chair Stand

**Figure 3:**
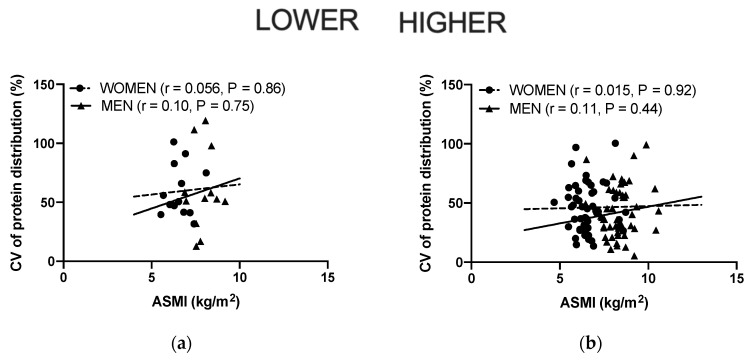

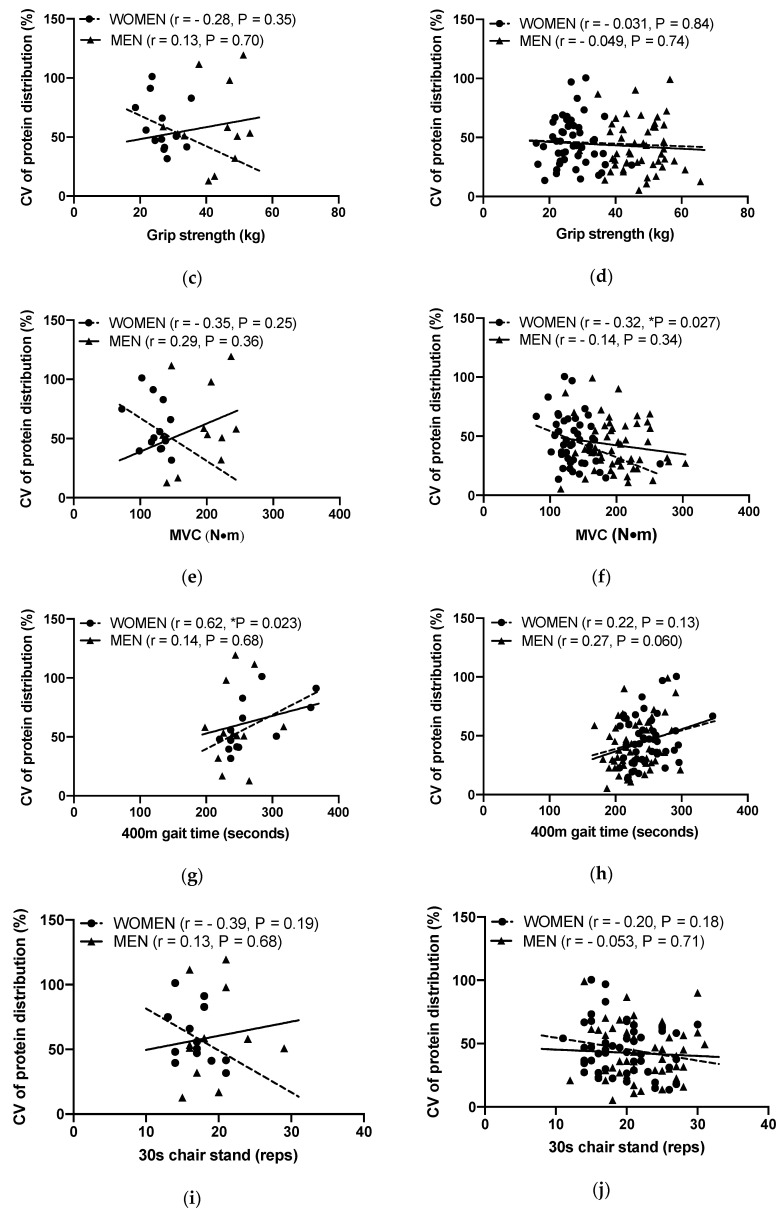
The association between the protein distribution for participants with a lower protein intake (*n* = 25, 12 men and 13 women) and a higher protein intake (*n* = 98, 50 men and 48 women (*n* = 47 for Maximal Voluntary isometric Contraction (MVC) due to one missing value)) and Appendicular Skeletal Muscle Index (ASMI) ((**a**) /Lower, (**b**) /Higher), grip strength ((**c**) /Lower, (**d**) /Higher), knee extension MVC ((**e**) /Lower, (**f**) /Higher), 400 m gait time ((**g**) /Lower, (**h**) /Higher), and 30 s chair stand ((**i**) /Lower, (**j**) /Higher). * *p* < 0.05, Pearson’s correlation coefficient was used to identify the associations. Significance was set at *p* < 0.05.

The association between the distribution of protein intake and functional abilities was examined separately for the participants ingesting lower amounts of protein (<0.83 g/kg aBW/day) and those ingesting higher amounts of protein (≥1.1 g/kg aBW/day) (Figure 3). Independent of the level of protein intake, the distribution was not associated with any of the functional measures (*p* > 0.05), except for MVC, where for women ingesting the highest level of protein there was a significantly higher MVC at the lowest CV values (*p* = 0.03, r = −0.32) for women ingesting the lowest level of protein, there was a significantly faster 400 m gait speed at the highest CV values (*p* = 0.02, r = 0.62).

### 3.5. Associations between Protein (g/kg aBW) and Energy (kJ/kg aBW) Intake

For total daily intakes and individual main meals at breakfast, lunch, and dinner, protein intakes were positively associated with energy intakes irrespective of sex (*p* < 0.0001) (Table 2).

## 4. Discussion

In the present study, we investigated the association between the total and meal distribution of protein and energy intake during the day, and the muscle mass, strength and physical function in healthy and well-functioning older Danish men and women. No association with the ASMI, grip strength, knee extension MVC or 400 m gait time appeared when dividing participants into three groups based on their protein intake (<0.83; ≥0.83–<1.1; ≥1.1 g/kg aBW), comparing the lowest and the highest of these three groups, or when correlating the protein meal distribution pattern. This suggests that within a population of healthy Danish older adults, neither the total protein intake nor meal distribution appear to be associated with muscle mass, strength or physical functions. Our data do not strengthen the emerging hypothesis that older adults need more protein than what is currently recommended to maintain muscle mass and physical function [57,58,59].

On average, the participants ingested more protein than 0.83 g/kg BW/day as recommended by the EFSA [7] and WHO/FAO/UNU [8]. However, there were no differences between those ingesting more than the recommendations and those ingesting less protein (Figure 2) when the total protein intake (<0.83; 0.83–<1.1; ≥1.1 g/kg aBW) and ASMI (Figure 2a) and various functional measures (Figure 2b–e) were compared. Similarly, Bollwein and colleagues found that among German seniors above the age of 75 years, daily protein intake was relatively high and did not make up any risk factor for frailty, and it was not evident even when they compared the highest and lowest quantile of protein intakes [48].

Protein intake pattern across the day, in addition to the total protein intake, has been suggested as an important factor for protein turnover and muscle mass [19]. A possible link between meal distribution pattern and benefits for muscle mass maintenance was demonstrated in an acute experimental study in healthy adults (36.9 ± 3.1 years) by Mamerow et al. [17]. They reported that the 24 h fractional synthesis rate is higher in an even protein distribution pattern (breakfast: lunch: dinner = 30: 30: 30 g) compared to a skewed distribution pattern (breakfast: lunch: dinner = 10: 15: 65 g). In a cross-sectional study, Bollwein and colleagues observed that the individuals in the frailest group (75–96 years) had a more skewed protein distribution pattern (less at breakfast and more at lunch) than those in the least frail group (76–91 years). It should be noted that neither group had an even distribution [48]. Supporting this notion, an observational study in adults (50–85 years) reported by Loenneke et al. [20] showed that those ingesting two meals or more containing above 30 g protein had a higher lean mass than those ingesting one meal or less containing 30 g protein. Similarly, in a two year follow up study in a 67–84 year old Canadian population by Farsijani et al. [16], those categorized as having the most even protein intake distribution pattern determined as the CV had a higher lean mass throughout the study regardless of total protein intake (~1.1 g/kg BW/day). In contrast, the decline in lean mass [16] and physical function [60] over the two-year period did not differ between protein intake patterns. Likewise, Kim and colleagues reported that total protein intake, but not the intake pattern, is responsible for the achievement of greater whole-body protein net balance in a study where a mixed macronutrient diet was provided in healthy older adults (52–75 years) [31]. They further supported the finding in an 8-week randomized controlled study that protein intake patterns in the context of a mixed macronutrients meal across the day is not a determinant of whole-body protein anabolism, MPS, muscle mass, and muscle function when an average amount of protein is consumed (i.e., 1.1 g/kg/day) in healthy older adults (51–69 years) [61].

In the present study, the participants were divided into higher and lower protein intake groups in order to investigate the association between the protein distribution (CV) and ASMI and physical performance parameters (Figure 1 and Figure 3). The use of the CV was adopted to isolate the distribution of protein, and not the amount of protein, as a variable. For example, it would be difficult to compare a skewed protein intake over the three main meals of 30, 30 and 60 g (120 g in total) with an even intake of 15, 15 and 15 g (45 g in total), as recently discussed by Hudson et al. [62]. It is evident, despite the limited number of participants, especially in the lower protein/energy intake groups, that in this population the protein distribution is not associated with the ASMI nor physical function. This is in line with a cross-sectional study conducted by Gingrich et al. [63], in which 97 community-dwelling older individuals were included (a mean age of 77 years). The divergent findings between our results, the study by Gingrich et al. [63], and other studies [16,20] could at least in part be explained by the different study populations that were investigated. Ten Haaf et al. [18] showed in a group of older individuals (≥65 years) that those having an even distribution of protein throughout the day were also the individuals who stayed the most active. This might explain why a population consuming protein evenly throughout the day, at least in some studies, had a higher lean mass.

Whole-body protein metabolism is influenced by energy intake [27,39,40] and energy balance [41]. Thus, the fate of utilization of amino acids for protein synthesis or energy production depends partly on total energy intake and energy balance. We found that protein and energy intake are positively correlated (Table 2), revealing that the participants ingesting high amounts of protein consume more energy in general, including protein and other macronutrients as part of their habitual dietary patterns. This is in accordance with observations in community-dwelling, frail and institutionalized elderly people as reported by Tieland et al. [64] and Smeuninx et al. [65]. The causality here cannot be determined, but we speculate that people with a high LBM and body mass may just eat more, leading to a concomitant increase in protein intake.

The phenotype observed in cross-sectional studies (lean body mass underlying the ASMI, muscle and grip strength, gait speed, etc.) is supposedly formed as a consequence of living conditions over several years. An underlying assumption for associating these phenotypic characteristics with food intake is, therefore, that the food recordings are representative of the long-term retrospective habitual food intake. The food questionnaires allowed us to explore this assumption. We found that 85 (46%) out of the total cohort mentioned that their food intake had changed after being retired. Of those, 21 (11%) directly stated that they had become more aware of eating a healthier diet, whereas nine (5%) stated that they were eating less and consumed easier foods (i.e., quick snacks, ready meals and fast food), snacked more and had become more relaxed towards healthy food choices. Twelve (7%) said that they went from being used to catering foods in excess at their workplace to more moderate and simple foods at home. In support for a transition in food intake occurring at retirement, a change in daily energy intake patterns was found among middle-aged British adults over a 17-year period, directed away from lunch and toward the evening meal [66]. Although it is not directly comparable to our population, this finding combined with our food questionnaires suggest that a change in food choices and intake pattern could be expected. Hence, longitudinal intervention studies should be conducted to account for such unavoidable uncertainty in a cross-sectional study design and investigate whether different protein intakes and dietary protein distributions preserve muscle mass and physical function in healthy older individuals.

A cross-sectional study design brings some limitations—as discussed above—and the sample size was relatively small in our study, especially in the lowest protein intake group (<0.83 g/kg/day). Further, the representative nature of a 3-day dietary registration and a 4-day physical activity registration may be questionable and thus creates some degree of uncertainly.

## 5. Conclusions

In conclusion, we found no associations between the protein and energy intake or distribution and ASMI and physical parameters in a cohort of healthy Danish older individuals. These data do not provide an incentive to recommend healthy, well-functioning older individuals who already adhere to the current internationally recommended dietary protein intake (0.83 g/kg/day) to change their dietary protein intake or distribution pattern throughout the day.

## Figures and Tables

**Table 1:** Participant characteristics for all, and divided into sex for higher (≥1.1 g/kg aBW/day) and lower protein intakes (<0.83 g/kg aBW/day).

	All (*n* = 184)	Women Lower (*n* = 13)	Women Higher (*n* = 48)	Men Lower (*n* = 12)	Men Higher (*n* = 50)	*p* Women/Men
Age _(years)_	70.2 ± 3.9	71.7 ± 4.1	71.0 ± 4.0	71.8 ± 5.7	68.9 ± 3.5	0.52/0.04
Age range _(years)_	65–82	65–80	65–81	66–82	65–78	
Height _(m)_	1.72 ± 0.10	1.65 ± 0.07	1.66 ± 0.06	1.79 ± 0.06	1.76 ± 0.06	0.93/0.30
Body Weight _(kg)_	74.9 ± 12.1	69.6 ± 7.7	65.4 ± 11.4	78.7 ± 7.2	79.0 ± 11.8	0.19/1.00
BMI _(kg/m_^2^_)_	25.4 ± 3.7	25.6 ± 4.0	23.8 ± 3.9	24.6 ± 2.2	25.4 ± 3.5	0.13/0.61
aBW _(kg)_	73 ± 8.7	67.5 ± 3.1	65.2 ± 7.0	78.4 ± 6.5	76.6 ± 7.6	0.23/0.44
WB LBM _(kg)_	48.5 ± 8.6	39.9 ± 2.6	40.2 ± 4.2	54.5 ± 4.3	55.0 ± 5.3	0.76/0.81
App. LBM _(kg)_	22.4 ± 4.6	18.2 ± 3.0	18.3 ± 2.0	25.4 ± 2.1	26.1 ± 3.3	0.84/0.43
EI _(MJ/day)_	8.2 ± 2.1	6.0 ± 1.3	8.5 ± 1.8	6.3 ± 1.6	9.7 ± 2.0	<0.001/<0.001
Protein _(Energy %)_	17.6 ± 4.0	14.3 ± 2.6	18.7 ± 4.8	15.9 ± 5.4	18.6 ± 3.1	<0.01/0.03
Protein _(g/day)_	82.8 ± 22.2	49.0 ± 8.5	90.6 ± 16.7	55.2 ± 10.2	104.3 ± 17.9	<0.001/<0.001
Protein _(g/kg BW/day)_	1.13 ± 0.34	0.70 ± 0.11	1.41 ± 0.30	0.70 ± 0.11	1.34 ± 0.25	<0.001/<0.001
Protein _(g/kg aBW/day)_	1.15 ± 0.31	0.73 ± 0.12	1.39 ± 0.25	0.70 ± 0.11	1.37 ± 0.23	
Goldberg Score						
EI/BMR	0.96 ± 0.24	0.77 ± 0.14	1.10 ± 0.24	0.65 ± 0.17	1.00 ± 0.19	<0.001/<0.001
Underreporters, *n*	41,(22%)	8,(53%)	4,(8%)	11,(85%)	5,(11%)	
Overreporters, *n*	18,(10%)	0,(0%)	9,(19%)	0,(0%)	6,(13%)	
Physical activity						
Step counts _(Steps/day)_	9740 ± 4358	9598 ± 3600	10,723 ± 4232	10,059 ± 5771	9297 ± 3392	0.036/0.30

aBW: adjusted body weight, BMI: body mass index, WB LBM: whole body lean body mass, App LBM: appendicular lean body mass, EI: energy intake, BMR: basal metabolic rate. The higher and lower protein intake differences of participant characteristics were compared using an unpaired t test. Significance was set at *p* < 0.05.

**Table 2:** Associations between protein (g/kg aBW) and energy (kJ/kg aBW) intake.

	Total		Breakfast		Lunch		Dinner	
	r	*R* ^2^	r	*R* ^2^	r	*R* ^2^	r	*R* ^2^
Women	0.69	0.48	0.72	0.52	0.82	0.68	0.56	0.32
Men	0.70	0.49	0.89	0.79	0.74	0.55	0.61	0.37

For all associations *p*-values are <0.0001.
